# Insights into the growth of nanoparticles in liquid polyol by thermal annealing†

**DOI:** 10.1039/d1na00222h

**Published:** 2021-06-28

**Authors:** Adrien Chauvin, Anastasiya Sergievskaya, Anna Fucikova, Cinthia Antunes Corrêa, Jozef Vesely, Jérôme Cornil, David Cornil, Milan Dopita, Stephanos Konstantinidis

**Affiliations:** Department of Condensed Matter Physics, Faculty of Mathematics and Physics, Charles University Ke Karlovu 5 121 16 Praha 2 Czech Republic adrien.chauvin@umons.ac.be; Chimie des Interactions Plasma-Surface (ChIPS), University of Mons Place du Parc 20 7000 Mons Belgium; Department of Chemical Physics and Optics, Faculty of Mathematics and Physics, Charles University Ke Karlovu 5 121 16 Praha 2 Czech Republic; Institute of Physics of the Czech Academy of Sciences Cukrovarnická 10/112 162 00 Prague 6 Czech Republic; Department of Physics of Materials, Faculty of Mathematics and Physics, Charles University Ke Karlovu 5 121 16 Praha 2 Czech Republic; Laboratory for Chemistry of Novel Materials (CMN), University of Mons Place du Parc 20 Mons 7000 Belgium

## Abstract

We report on the growth of metal- and metal-oxide based nanoparticles (NPs) in heated polyol solutions. For this purpose, NPs are produced by the sputtering of a silver, gold, or a copper target to produce either silver, gold, or copper oxide NPs in pentaerythritol ethoxylate (PEEL) which has been annealed up to 200 °C. The objective of the annealing step is the fine modulation of their size. Thus, the evolution of the NP size and shape after thermal annealing is explained according to collision/coalescence kinetics and the affinity between the metal-/metal-oxide and PEEL molecule. Moreover, highlights of few phenomena arising from the annealing step are described such as (i) the reduction of copper oxide into copper by the polyol process and (ii) the effective formation of carbon dots after annealing at 200 °C.

## Abstract 2

The abnormal growth of metal and metal-oxide nanoparticles under thermal treatment in a polyol solution is explained. The process also allows the catalysis of carbon dot formation and the reduction of copper oxide.

## Graphical abstract

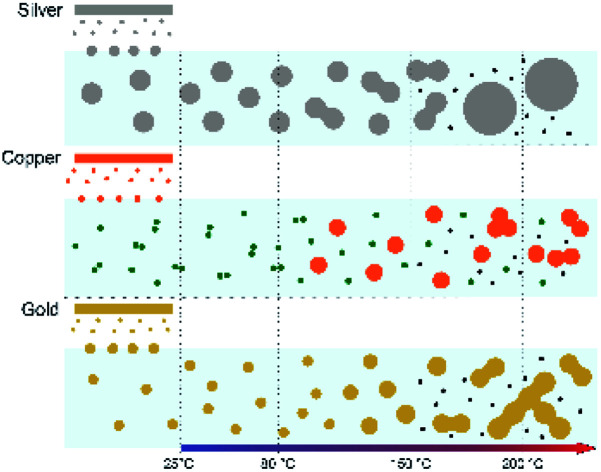

## Introduction

Owing to the progress made in nanoscience and nanotechnology, the bottom-up approach involving the assembly and growth of NPs has been a topic of both scientific and technological importance. The predominant process underlying the growth of NPs relies on the coalescence mechanism that can occur at various stages during material processing, *i.e.*, during synthesis itself or after the synthesis, through controlled thermal annealing.^1^ Coalescence of NPs, where two or more particles are attached and merge into a bigger one, often arises from their instability. From a thermodynamics point of view, NPs tend to coalesce to reduce the total surface energy due to their large surface-to-volume ratio.^2^ The coalescence is considered as a thermally activated, capillary driven, and continuous process, which starts with the collision of NPs and the formation of a neck connecting these NPs.^3,4^ Then, thanks to atomic surface diffusion, the material flows from each NP towards the neck binding them.^5^ This surface diffusion mechanism leads to the formation of fused NPs.^6^ Finally, grain growth takes place by volume diffusion, so that the deformation and the twin fault densities decrease.^7^ The coalescence of a collection of NPs follows the Smoluchowski coagulation kinetics which predict a power-law increase of the NP sizes and size dispersion with time.^8,9^ Understanding the coalescence mechanism of NPs has allowed gaining control on growth rates and NP sizes and shapes through the selection or tuning process parameters.^10^ Despite the advances in the understanding of NP formation, this coalescence mechanism alone cannot thoroughly explain the growth of NPs in solutions.^2,11^ In a majority of NPs grown in solutions, the surfaces of NPs are usually functionalized with organic ligands.^12^ Thus, in the case of stabilized NPs, the growth during annealing is driven by a two-stage process: an initial stage, wherein growth is mostly due to Ostwald ripening (OR), and a later stage characterized by the growth by coalescence similar to the case of unstabilized NPs.^8^ In OR, the NP growth is driven by the transfer of atoms from smaller NPs to larger ones, making it a one-way transfer, while in coalescence both NPs provide material.^8^ The transition stage between these two mechanisms, in the case of stabilized NPs, is highlighted by the desorption of the molecules from the NP surface.^8,13^

In ideal cases, the NP growth leads to larger and spherical NPs. However, this process is not always straightforward. The formation of anisotropic structures can be observed during the coalescence step. Briefly, the coalescence is driven by the collision time (*τ*_collision_), corresponding to the time needed for the collision between NPs, and the coalescence time (*τ*_coalescence_), corresponding to the time needed for two NPs to be fully fused into one. These two parameters are associated with the annealing temperature and the size of the NPs.^14,15^ Assuming that *τ*_collision_ follows the Brownian collision model and *τ*_coalescence_ is driven by the grain-boundary diffusion, when the size of the NPs increases, *τ*_collision_ and *τ*_coalescence_ decrease.^16,17^ Moreover, *τ*_coalescence_ is dependent on the element constituting the NPs since the melting temperature and the surface diffusion vary from an element to another.^15^ Thus, if the NPs coalesce faster than they collide (*τ*_collision_ ≫ *τ*_coalescence_), collisions of NPs will result in large spherical NPs. However, if NPs' coalescence is negligibly slow (*τ*_collision_ ≪ *τ*_coalescence_), a collection of small attached particles (aggregates) is produced.^8,13,18^

With the development of an innovative synthesis approach leading to the production of NPs dispersed in solutions, namely sputtering onto liquids, it becomes essential to look into the growth mechanisms of NPs during annealing in liquid media. Sputtering onto liquids allows the production of well crystallized, small, and pure metal NPs varying from a few angstroms to few tens of nanometers in radius.^19,20^ The tuning of the sizes of the so-produced NPs during the synthesis can be done by the use of liquid substrates characterized by different viscosities.^21^ However this approach is limited and restrained to a small range of diameter of NPs. The limitation of this approach lies in the critical viscosity from which the creation of a film at the surface of the liquid begins.^21^ Moreover, this approach is restrictive since the diameters of the NPs vary only from 1.8 to 2.3 nm.^21^ A successful approach reported a few years ago relies on heating the solution to induce the growth of the NPs into bigger ones.^22^ Despite the observation of the NP growth, no insights into the actual mechanism of the process were provided.

In the present article, we discuss the growth behaviour of NPs obtained by magnetron sputtering of pure metals onto liquid polyol. The sputtering of a gold, copper, or a silver target was performed to obtain highly dispersed pure gold, copper oxide, or silver NPs in pentaerythritol ethoxylate (PEEL). PEEL is an organic oil used as a substrate and NP host matrix. PEEL is able to withstand the low pressure during the deposition and it is easier to handle than ionic liquids commonly used for this synthesis approach. PEEL solutions containing the NPs were annealed to monitor their growth behaviour.

## Results and discussion

### Selection of the elements

Three materials are selected for this study, *i.e.* copper, gold, and silver. On the one hand, from thermodynamics, it is known that gold is inert, with an enthalpy of oxidation equal to +19.3 kJ mol^−1^.^23^ On the other hand, quantum chemistry calculations show that, the interaction of the PEEL molecule with gold is favourable (Table 1). Thus, the formation of metallic gold NPs is expected in PEEL.^24^ In contrast, silver, and copper are more easily oxidized. Indeed, the enthalpy of formation of Ag_2_O and Cu_2_O is negative, *i.e.*, −31.1 kJ mol^−1^ and −168.6 kJ mol^−1^, respectively.^25,26^ In spite of the negative enthalpy of silver oxide formation, a previous study reports the formation of metallic silver NPs after sputtering onto PEEL.^25^ Thus, silver NPs are expected in the solution after sputtering. In contrast, a previous study shows the formation of copper oxide NPs after sputtering of copper onto liquids after few days of storage.^26^ In this study, annealing of solutions and characterization were carried out a few days after the synthesis of the NPs. The presence of copper oxide NPs is thus expected. Moreover, the DFT calculations (Table 1) reveal a negative absorption energy for all the metal- and metal-oxide NPs. Thus, the PEEL molecules act as a stabilizer for the NPs.

**Table 1:** Interaction energies, charge transferred, and total bond order between the model PEEL molecule and the different surfaces, as calculated at the Density Functional Theory (DFT) level

Surface	Facet	Adsorption energy (eV)	Charge on the molecule (|*e*|)	Total BO
Au	111	−0.22	0.27	0.69
	110	−0.32	0.22	0.62
	100	−0.27	0.20	0.70
Ag	111	−0.15	0.11	0.91
	110	−0.26	0.15	0.58
	100	−0.12	0.17	0.62
Cu	111	−0.29	0.26	0.95
	110	−0.34	0.23	0.93
	100	−0.44	0.24	0.82
Cu_2_O	111	−1.71	0.24	1.62
	110	−1.08	0.21	1.63
	100	−2.53	0.21	2.03

### Characterization approach

**Fig. 1:**
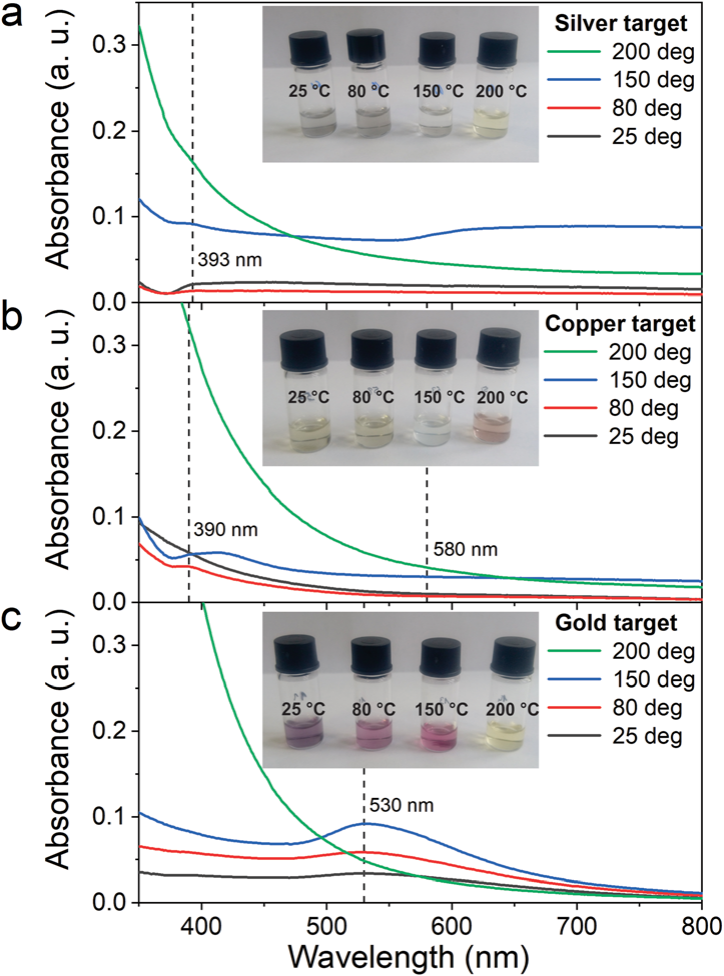
Absorption spectra of diluted PEEL solution after sputtering of the (a) silver target, (b) copper target, (c) and gold target in their pristine state (black curve) and after annealing at 80 °C (red), 150 °C (blue) and 200 °C (green) for 5 h. The inset corresponds to the PEEL solution containing the NPs diluted in the same way for all the samples in ethanol for the different annealing temperatures.

**Fig. 2:**
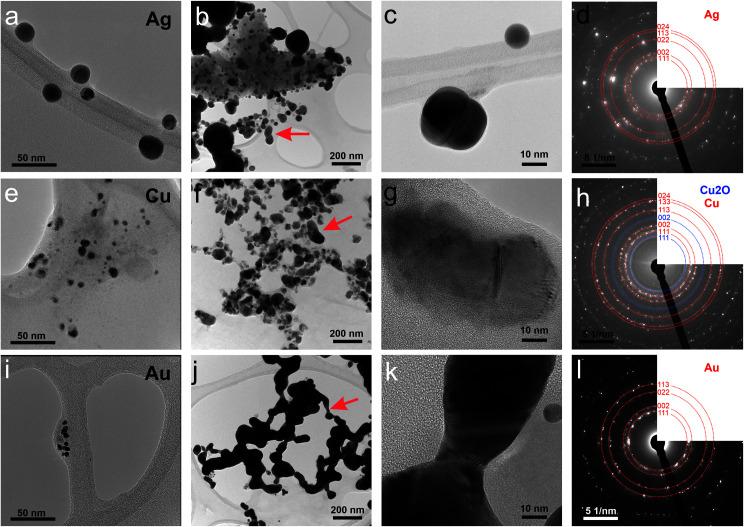
Transmission electron micrographs of (a–c) silver, (e–g) copper/copper oxide and (i–k) gold NPs (a, e and i) without annealing and after annealing at 200 °C for 5 h at (b, f and j) low and (c, g and k) high magnification. Electron diffraction spectra of selected areas after annealing at 200 °C for 5 h of (d) silver, (h) copper/copper oxide, and (l) gold NPs. Arrows in panels (b), (f) and (j) highlight the neck between NPs.

**Fig. 3:**
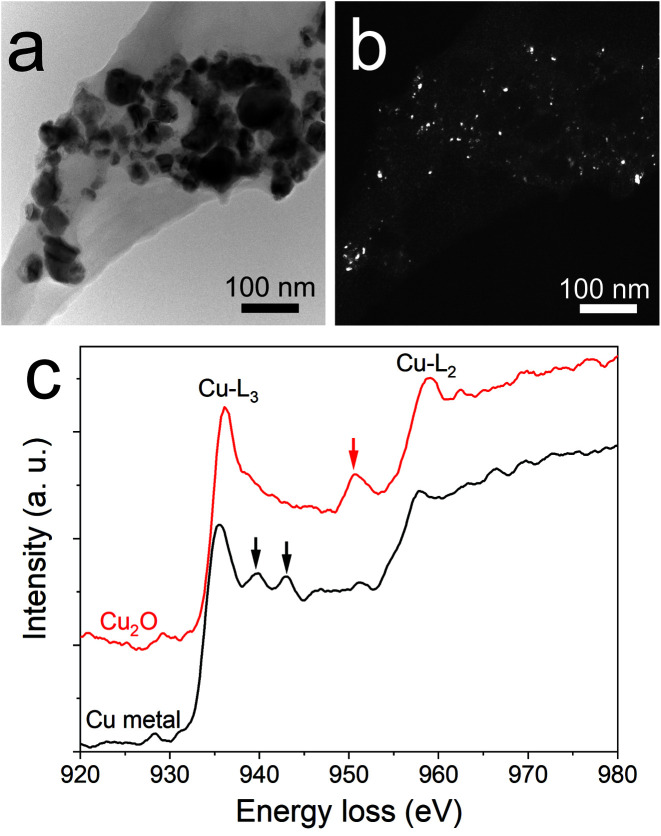
(a) Bright field image of copper/copper oxide after annealing at 200 °C for 5 h. (b) Dark field image from the (200) ring of Cu_2_O representing the same area as in panel (a) and (c) electron energy loss spectroscopy (EELS) of Cu-L_2,3_ in two areas.

**Fig. 4:**
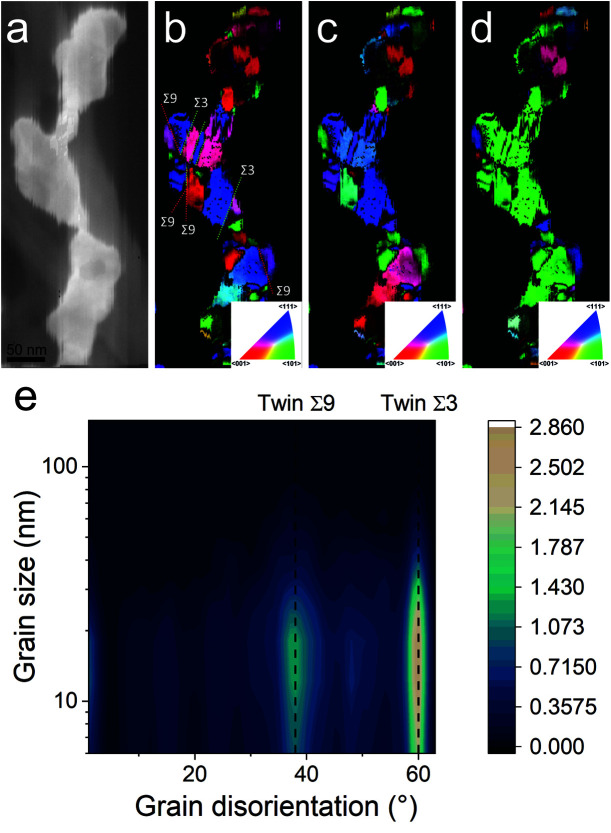
(a) Scanning TEM images and (b–d) crystal orientation mapping of an elongated structure observed for annealed gold NPs at 200 °C for 5 h along the (b) *X*, (c) *Y*, and (d) *Z* directions. (e) Calculated grain disorientation as a function of the grain size, revealing the *Σ*9 and *Σ*3 twins (highlighted in panel (b)).

**Fig. 5:**
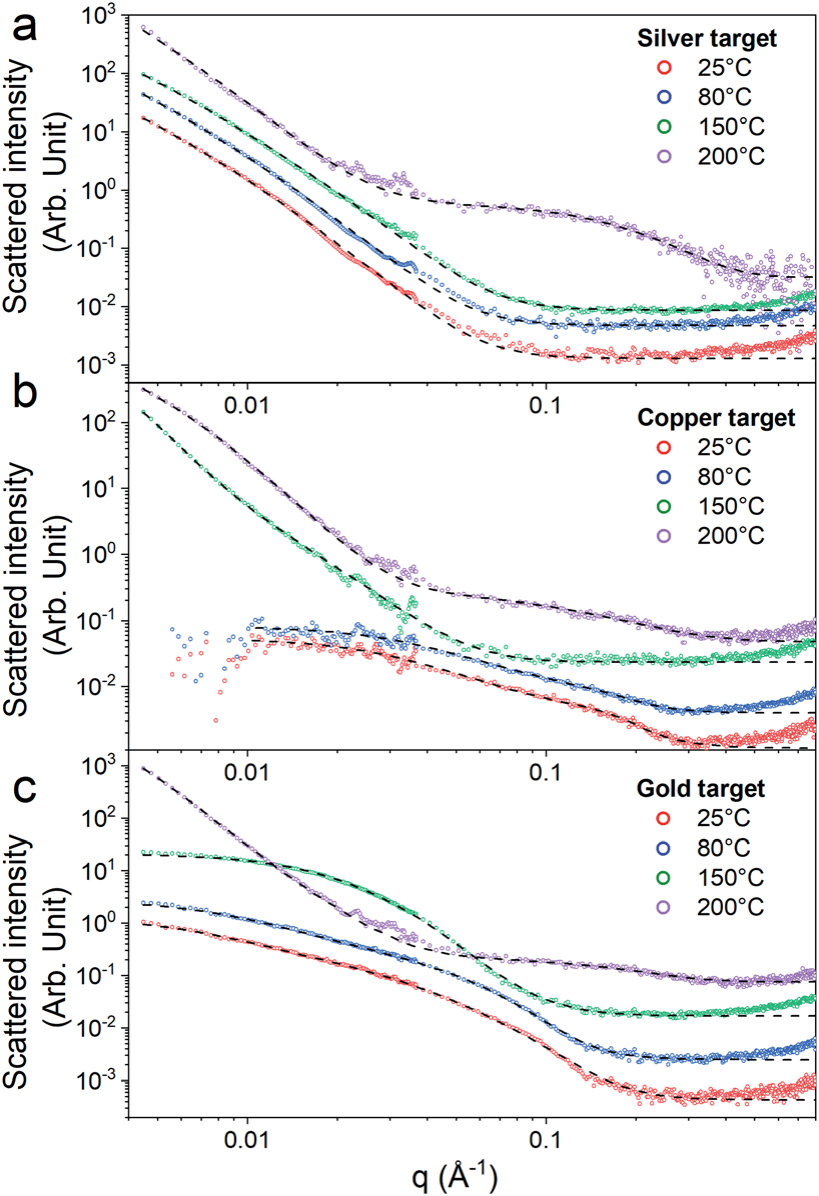
SAXS patterns (dots) and the resulting IRENA fit (dashed line) of PEEL solutions containing (a) silver, (b) copper, and (c) gold NPs after sputtering (purple) and after annealing at 80 (red), 150 (blue) and 200 °C (green) for 5 h. The curves are shifted along the *Y*-axis for better visualization.

First, the optical properties of the solutions were considered. The naked-eye observation of the plasma-treated PEEL solutions was followed by UV-vis absorption spectrophotometry studies for the different annealing temperatures (Fig. 1). Then, to get a better insight into the evolution of the morphology, TEM analysis of the NPs was performed before and after their coalescence at 200 °C (Fig. 2–4). Since TEM analyses are performed after removing the annealed solution, SAXS analysis was also performed to complete the study, *i.e.* by studying the NPs inside the solution. The *in situ* SAXS measurements were done on the pristine (*i.e.*, not annealed) and annealed PEEL solutions containing the NPs. The resulting patterns are displayed in Fig. 5. The same procedure as described in our previous paper was used to fit the SAXS patterns.^27,28^ First, for all SAXS patterns, in the high *q* part, the slope of *q*^−4^ indicates that the surface of the structure is smooth.^29^ The average radius and the dispersion of the NPs were evaluated by fitting the scattering data using dedicated software, *i.e.*, IGOR Pro implemented with the IRENA package.^30^ In most cases, the diluted model was used, pointing the absence of interactions between the NPs in the solution. However, for the solution containing the Cu_2_O NPs at ambient temperature and 80 °C, the hard-sphere model was needed. In contrast to the dilute model, the latter highlights the interaction of the NPs. The hard sphere model is characterized by the hard-sphere radius *R*_HS_ corresponding to the correlation distance of the particles within an aggregate and the mean hard-sphere volume *η* describing the number of NPs in an aggregate.^31^ The hypothesis drawn by the characterization approach is supported by DFT calculations. The adsorption energy of a model molecule, *i.e.* pentaerythritol molecule (C(CH_2_OH)_4_), was calculated for different facets, *i.e.* (111), (110) and (100), for each metal- and metal-oxide NP reported in this study (Table 1).

**Fig. 6:**
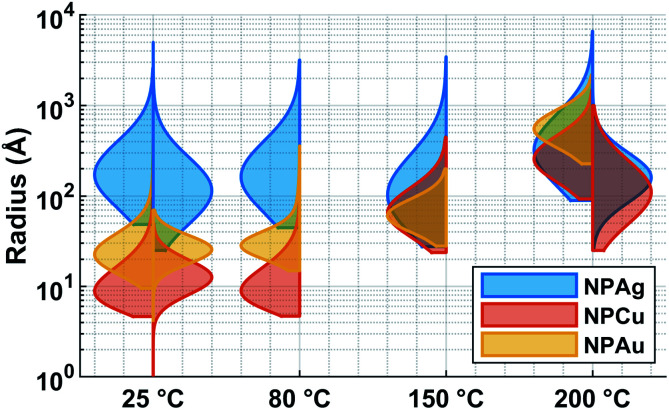
Violin plot of the size distribution of the NPs in the PEEL after sputtering (25 °C) and after annealing at 80 °C, 150 °C and 200 °C for the solution containing the silver NPs (blue), copper NPs (orange), and gold NPs (yellow) obtained by SAXS analysis (left) and by TEM (right).

**Fig. 7:**
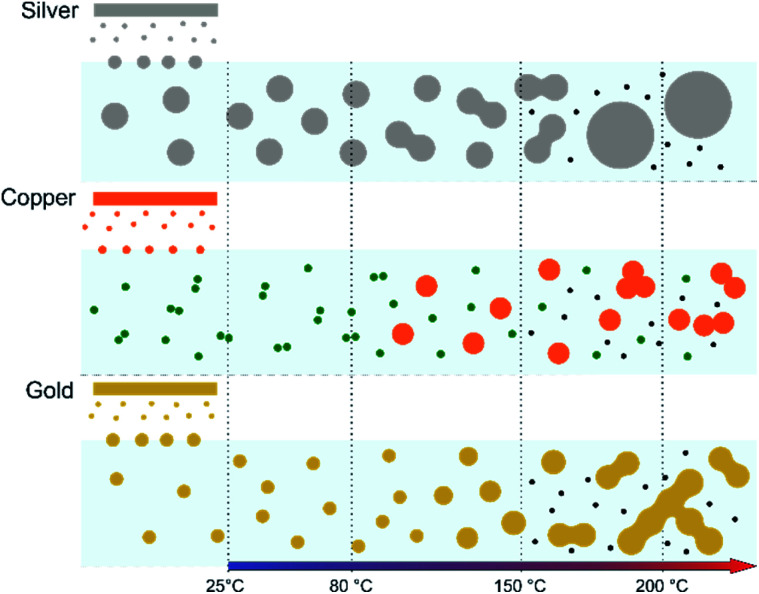
Scheme of the structural evolution of the NPs during annealing in PEEL after sputtering of silver, gold and copper targets. Grey, yellow, orange, green and black dots represent silver, gold, copper, copper oxide and carbon NPs, respectively.

The characterization conducted on the solutions before and after thermal annealing for a variety of temperatures allows us to get a better picture of the NP growth mechanism and the stability of the NPs inside the PEEL under thermal treatment (Fig. 6 and 7). First, the influence of the heating treatment on each metal and metal-oxide solutions is examined. Then, the global coalescence behaviour is discussed.

### Silver

In the case of silver, the solution exhibits a grey colour after deposition with visible sedimentation after one month of storage in air (Fig. 1a). Moreover, NPs have a circular shape with a mean radius of 16 ± 8 nm as highlighted by the TEM analysis (Fig. 2a) and ∼18 nm by SAXS analysis (Fig. 5a). Upon annealing up to 80 °C, the UV-vis absorption spectrum of silver NPs shows only a weak and broad surface plasmon resonance (SPR) peak at 393 nm (Fig. 1a). It is worth noting that there is no SPR peak characteristic of silver, even though NPs having a mean radius of a few tens of nanometers were detected by TEM and SAXS (Fig. 6).^20,32^ This behaviour might be due to the aggregation of NPs, which can hinder the characteristic SPR peak of metal NPs.^33^ After further annealing at 150 °C, elongated metallic NPs are evidenced by UV-vis analysis with the appearance of a second SPR peak at higher wavelength (Fig. 1a).^32,34^ Thus, at 150 °C, the desorption of PEEL takes place and the collision of the Ag NPs increases. However, due to the rather low temperature, *τ*_coalescence_ is very slow compared to *τ*_collision_, which leads to the creation of anisotropic structures. Moreover, the difference in adsorption energies between the different facets of silver (Table 1) can induce the partial desorption of PEEL from the surface characterized by the weakest bonding and the coalescence in the form of elongated structures. Elongated structures, like two NPs connected by a neck, are the result of incomplete coalescence.^35^ The creation of elongated structures at 150 °C is not detected in the SAXS pattern (Fig. 3a) because these structures might be too large to be detected by our SAXS device. Finally, after annealing at 200 °C, the NPs still exhibit a metallic crystalline behavior as seen from the SAED patterns (Fig. 2d) and a spherical shape with only a few elongated structures left for observation on the TEM grid (Fig. 2b and c). Moreover, a linear increase in the mean NP radius is reported, from ∼18 to ∼45 nm by SAXS and from 16 to 41 ± 37 nm by TEM (Fig. 6). It is important to stress here that the large standard deviation from the TEM analysis reveals a broad distribution in the sizes of the NPs (Fig. 6).^22^ This highlights the increase of *τ*_coalescence_ together with the weakening of the bond between PEEL molecules and all facets of the silver NPs. According to the calculations, the adsorption energy of PEEL over the silver surface is lower than for the copper and gold surfaces (Table 1), so that less energy is needed to weaken the PEEL–Ag bonding. Thus, the coalescence of silver NPs is less restrained compared to copper and gold (Fig. 7).

### Copper

The solution containing the copper NPs is yellowish at 25 °C (Fig. 1b). This colour is related to the formation of copper oxide NPs.^26,27^ This is consistent with the literature report on the oxidation of copper during storage after sputtering onto PEEL.^26,27^ In terms of size, the copper NPs exhibit a size significantly lower than that of the silver NPs with a mean radius of 1.2 ± 0.8 nm measured by TEM (Fig. 2e) and 1.1 nm by SAXS (Fig. 5b). The presence of small NPs is supported by the absence of the SPR peak in the UV-vis absorption spectra (Fig. 1b). The SAXS pattern of NPs obtained after sputtering reveals the interaction between NPs. The fitting curves of the SAXS pattern yield a mean radius of 1.1 nm with an *R*_HS_ of 1.6 nm. The small *R*_HS_ compared to the mean radius indicates that the NPs gather in a small cluster of 2 or 3 NPs. For an annealing temperature of 80 °C, the same behaviour is observed in the SAXS pattern (Fig. 5b) and a small SPR peak at ∼390 nm is observed in the UV-vis absorption spectra (Fig. 1b), which can be linked to a better dilution of the copper oxide NPs.

When an annealing temperature of 150 °C is reached, the UV-vis absorption spectrum (Fig. 1b) shows an SPR peak corresponding to copper oxide NPs.^36^ The peak at ∼390 nm is attributed to the band-to-band transition of Cu_2_O usually reported for small Cu_2_O nanocrystals ranging from 2 to 10 nm.^37,38^ The presence of a weak and wide peak at 580 nm is reported. It corresponds to the bandgap transition of CuO at the surface of the nanocrystal.^37,39^ Moreover, for the solutions annealed at 150 °C and 200 °C, larger NPs are observed by SAXS (Fig. 5b). The sudden change in slope between 80 °C and 150 °C points to the instability of copper oxide NPs over 80 °C and their fast growth. The pattern fitting reveals a mean radius size of 10 nm and 36 nm when increasing the annealing temperature from 150 °C to 200 °C, respectively. In these two cases, using a dilute model instead of a hard-sphere model used for lower annealing temperatures was required. At 150 °C, the fitting also reveals a slope of *q*^−4^ in the Guinier region which corresponds to the part where *q* < π/*R*.^40^ The linearity in the Guinier region highlights the dispersity of the NPs and suggests that weak interparticle interactions are present in the sample.^41^ Thus, this slope reflects the presence of ‘soft’ agglomeration of NPs. This ‘soft’ agglomeration of NPs enhances the scattering in the low *q* region with random behaviour due to the weak bonding between NPs.^42^ This feature is not observable for the NPs annealed at 200 °C due to the limitation in the probed *q*-range allowed by our device. The SAXS observations are coherent with the TEM analysis. The radius of the NPs is 19.6 ± 8.6 nm after annealing at 200 °C and they exhibit a spherical shape with the occurrence of fused NPs (Fig. 2f and g). The fused NP structure can be described as two particles connected by a neck (Fig. 2g). This structure is characteristic of NP coalescence.^43^

An interesting finding is obtained for the NP composition after annealing at 200 °C. SAED (Fig. 2h) revealed that the structure is mainly constituted of the crystalline FCC copper structure, though weak rings of Cu_2_O are also detected. The dark field image from the (002) ring of Cu_2_O highlights the location of Cu_2_O NPs (Fig. 3). It reveals that the Cu_2_O NPs (in white) are very small as compared to the metallic copper ones (in black). The observation of both Cu_2_O and metallic copper is further confirmed by electron energy loss spectroscopy (EELS) performed at the Cu-L edge (Fig. 3c). The two major features of these edges are the strong white-lines L_3_ and L_2_, due to the spin–orbit splitting of the 2p core hole, and separated by about 20 eV.^44^ The Cu-L_2,3_ edges of metallic copper exhibit a typical step-like shape with the appearance of two peaks close to the Cu-L_2_ edge. In Cu_2_O, the Cu-L_3_ edge presents a strong and asymmetric peak at the threshold and a peak close to the Cu-L_2_ edge. These peaks are marked by arrows in Fig. 3c. Moreover, the shape of the Cu-L_2_ edge is similar to that of the Cu-L_3_ edge.^44^

The presence of copper NPs after annealing is due to the reduction of copper oxide into copper referred to as the polyol process.^45,46^ This behavior has been reported in the literature for the annealing of copper oxide in ethylene glycol at 170 °C.^47^ In our case, the chemical reduction is enabled by the presence of PEEL. Indeed, PEEL contains four hydroxyl groups allowing for the reduction of the metal oxide.^47,48^ When looking at the dark field from the (002) ring of copper oxide (Fig. 3a and b), one can observe that the copper oxide NPs are small compared to the metallic copper NPs. This observation highlights the influence of PEEL adsorption over the NP surface on their growth. It is important to stress here that the calculated adsorption energy of PEEL (Table 1) is the highest for copper oxide (*i.e.*, −1.71 eV for 111 surfaces) compared to metallic copper NPs (−0.29 eV for 111 surfaces), so that more energy is needed to weaken the bond between the PEEL molecule and the surface.^7,49^ Thus, the temperature required to induce the coalescence of copper oxide NPs is higher than for copper NPs.

### Gold

The colour of the solution containing gold NPs evolves from violet after deposition (*i.e.*, no annealing performed) to pink upon annealing at 150 °C. This behavior reveals the nanoscale evolvement of the size of the NPs (Fig. 1c). Indeed, the size of the NPs increases from 3 nm in radius after sputtering to 7 nm after annealing at 150 °C (Fig. 4). In the UV-vis absorption spectra, within the same annealing range, an SPR peak is detected at 530 nm corresponding to the transverse plasmon resonance of gold NPs (Fig. 1c). The broadness of the peak reflects the aggregation of the gold NPs.^50^ Moreover, with the increase in annealing temperature, the transverse plasmon resonance grows in intensity gradually. This evolution is linked to the growth of the gold NPs when increasing the annealing temperature.^51^ A clear evolution of the SAXS pattern can be noticed here. First, for fitting the pattern at 25 °C and 80 °C (Fig. 3c), two populations must be involved. The first population has a mean radius of 3 nm and the second 5 nm. Then, after annealing at 150 °C, the fit of the SAXS pattern requires only one population with a mean radius of 7 nm, which is larger than the radius of NPs right after sputtering.

When the temperature reaches 200 °C, the behavior of the NPs changes. First, in the SAXS pattern (Fig. 5c), the size of the object in the PEEL appears to be very large and only a rough estimation of the mean radius size can be done. According to the fit of the SAXS pattern, the mean radius of the objects present in the PEEL solution is around 70 nm, *i.e.*, ten times higher than the mean radius observed after annealing at 150 °C (Fig. 6). Moreover, mesh-like structures are seen in the TEM images (Fig. 2j). The shape represents a network of NPs connected by a neck (Fig. 2k). In this case, many ligaments are connected to each other, creating a mesh-like structure. The SAED points to a well-crystallized structure (Fig. 2l).

A possible explanation for the formation of such a mesh-like structure is that (Fig. 7), until 150 °C, the gold NPs grow by OR. When the temperature reaches 200 °C, the partial desorption of PEEL from the gold NP facets and the orientated attachment drive the formation of elongated structures. The SAXS pattern supports the growth by OR since only one well-dispersed population is observed for the annealing at 150 °C, while, before 150 °C, two populations of spherical NPs are detected. Then, upon annealing at 200 °C, structures with a large radius are consistent with the rapid growth by collision and coalescence. As mentioned previously, when *τ*_collision_ is faster than *τ*_coalescence_, the resulting structure corresponds to an aggregate of NPs, as reported in the literature for stabilized gold NPs.^7^ This hypothesis is also supported by the automated crystal orientation mapping in TEM (ACOM-TEM) performed on the ligaments (Fig. 4) since the mean grain size is close to that reported for the size of the Au NPs after annealing at 150 °C (6.5 ± 2 nm in radius). Moreover, the adsorption energy between the molecule and each gold facet is different and higher than for silver (Table 2). Thus, the bond weakening is different for each facet and requires more energy than for silver NPs, resulting in the mesh-like growth.^48^ Finally, when looking at the orientation mapping analysis of a ligament (Fig. 4a–d), one can characterize the grain boundaries by their degree of fit (*Σ*) based on the coincidence site lattice (CSL) model (Fig. 4e). In a polycrystalline structure, two neighbouring grains share a common crystallographic plane. However, the grain boundary (GB) between them reveals the rotation of the grain according to its neighbour. This rotation called gain disorientation (or misorientation) can be highlighted by the misorientation mapping (Fig. 4e). By determining the coordinates of each grain through ACOM-TEM (Fig. 4b–d), the misorientation between them can be extracted and plotted through the frequency distribution of the grain disorientation between neighbouring grains in a structure (Fig. 4e). The parameter *Σ* defines the relationship between two grains and corresponds to the ratio between the total number of sites and the coincidence sites (*i.e.* number of shared atoms).^52^ The corresponding angle/axis and CSL GB *Σ*3 = 60°/〈111〉, (*i.e.* the rotation of neighbouring grains of 60° around the 〈111〉 axis) and *Σ*9 = 38.94°/〈101〉 are deduced and frequently encountered in the mesh-like structure. This observation matches the one already reported during the oriented attachment of the gold NPs and the growth of elongated structures.^12,52^

**Table 2:** Deposition conditions used in this study

	Silver	Copper	Gold
Power applied on the target (W)	20	75	17
Deposition speed (nm min^−1^)	11	15	5.1
Amount of sputtered material inside the liquid (mg cm^−3^)	2.6	3	2.2

### Global overview

A great asset of our study is the possibility to compare each metal behavior inside PEEL and rationalize the size of the NPs after sputtering. According to our study, the mean size of the NPs appears to be linked to the affinity of the metallic surface for the PEEL molecule. Indeed, the mean radius reported for metallic samples after sputtering evolves from 35, 3.2, and 1.4 nm for interaction energies obtained by DFT calculations equal to −0.15 (silver), −0.22 (gold), and −0.29 (copper) eV, respectively.^25,27^ Higher affinity of the metal for the PEEL leads to smaller NPs.

**Fig. 8:**
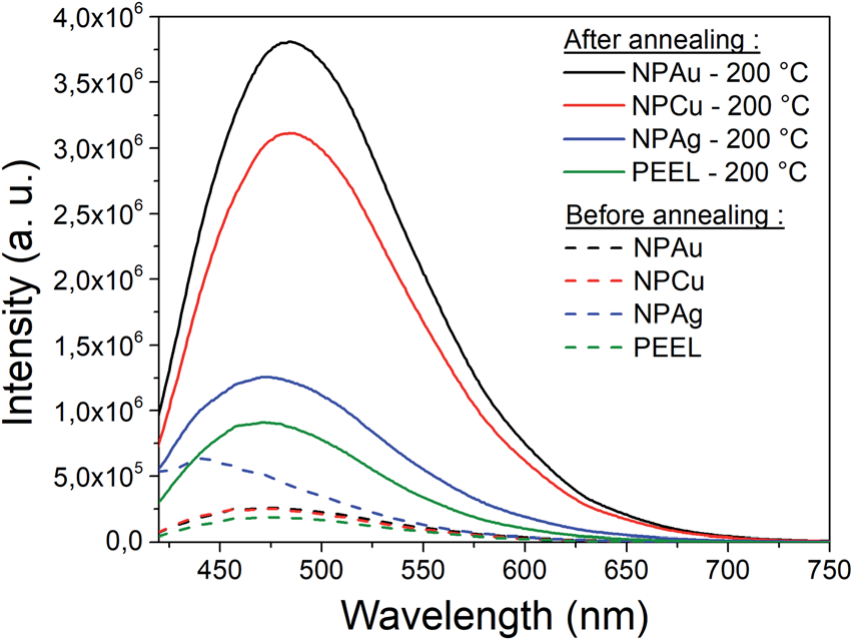
Photoluminescence of the solution before (dash line) and after (full line) annealing at 200 °C for the pristine PEEL solution (green) and PEEL solution containing silver NPs (blue), copper NPs (red), and gold NPs (black) at an excitation wavelength of 405 nm.

An attractive feature is also the appearance of the SAXS pattern related to small NPs, *i.e.*, whose mean radius equals 0.5 nm, after annealing all solutions containing the NPs at 200 °C (Fig. 5). These small NPs result from the decomposition of the PEEL molecules. Indeed, the thermal decomposition of polyol proceeds at about 50 °C below their boiling point and induces the formation of C-dots with a diameter of a few nanometers.^45^ The process leading to the formation of C-dots relies on the pyrolysis–clustering–carbonization which is assisted by the direct heating in air in our case.^45,53,54^ This hypothesis is further supported by the fact that UV-vis absorption spectra present a strong absorbance at low wavelength related to C-dots, for all samples annealed at 200 °C.^55^ Moreover, the yellow colour of the solution after annealing is proof of the presence of these C-dots.^55^ To validate this assumption, the photoluminescence (PL) of the solution has been recorded (Fig. 8). Indeed, it has been shown that the C-dots exhibit an intense blue to green fluorescence.^45^ First, the sputtering of the different targets onto PEEL increases the PL of the solution only slightly as compared to pristine PEEL. Then, an increase of the PL is detected for all the solutions after annealing at 200 °C, including pristine PEEL. This strong PL after annealing is the evidence of the presence of C-dots in the solution.^45^ Finally, a stronger PL signal for the solution containing the NPs and annealed at 200 °C is obtained as compared to the solution without NPs. This result suggests that the use of metallic NPs for the catalysis of the C-dot formation in a polyol solution is effective. Moreover, since NPs can be removed from the solution by centrifugation, this approach provides a good alternative to create pure C-dots. The C-dots can be further extracted and purified from the solution by filtration.

## Conclusions

In this work, the growth mechanism of different metal- and metal-oxide NPs in polyols is unravelled. The influence of heating the solution is also discussed. NPs have been synthesized by sputtering onto liquids. Three metal targets, *i.e.*, silver, copper, and gold, were sputtered onto PEEL leading to the formation of silver, copper oxide, and gold NPs, respectively. The different mean sizes observed in our samples can be explained by considering the affinity of the PEEL molecules to metal- and metal-oxide surfaces *via* quantum-chemical calculations. When thermal annealing of the solutions containing the NPs is performed up to 200 °C, the growth of the NPs is induced. Different morphologies are obtained for the NPs after annealing at 200 °C as a function of the precursor chemical nature. This observation is correlated with (i) their affinity for the surrounding media and (ii) the interplay between *τ*_collision_ and *τ*_coalescence_. For silver NPs, the low affinity for the PEEL molecules, together with *τ*_collision_ > *τ*_coalescence_, only allows for a slight growth of the NPs, which exhibit a spherical shape after annealing at 200 °C. This behaviour is also related to the bigger size of the silver NPs obtained after sputtering as compared to gold and copper oxide NPs, which slows down the *τ*_collision_ and *τ*_coalescence_ and leads to only a slight increase of the NPs. For a metal with almost the same affinity for PEEL, *i.e.*, for gold NPs, the formation of interconnected NPs was revealed due to the inversion of the two key timescales: *τ*_collision_ < *τ*_coalescence_. Finally, when the affinity for the PEEL molecules increases, *i.e.*, as for copper, the aggregation of the NPs with only a slight increase of the mean size is promoted due to OR. Besides the size and shape evolution of the NPs, other additional phenomena have been reported, such as the formation of C-dots during annealing and the reduction of the copper oxide NPs into copper through the polyol process.

This study provides better insight into the behavior of the NPs subjected to thermal annealing in a polyol and can be of interest for the effective production of C-dots and applications such as energy storage or in biology.^56,57^

## Experimental

### Synthesis of NPs

Pentaerythritol ethoxylate (PEEL; C[CH_2_(OCH_2_CH_2_)_*n*_OH]_4_, 15/4 EO/OH, *M* ∼ 797 g mol^−1^, from Sigma Aldrich) was stored without any specific precaution. The NPs were synthesized by direct current (DC) magnetron sputtering. Argon ions generated inside the argon plasma were used to sputter either a gold (diameter: 76.2 mm; purity: 99.99%), a copper (diameter: 76.2 mm; purity: 99.99%) or a silver (diameter: 76.2 mm; purity: 99.99%) target inside a dedicated vacuum chamber. For these sputtering experiments, the same procedure was used, *i.e.* 2.5 ml of PEEL was poured into a watch glass and the distance from the magnetron source was 130 mm. The electrical power applied to the silver, copper, and gold targets was fixed to 20 W (with a sputtering current of 60 mA), 75 W (with a sputtering current of 175 mA), and 17 W (with a sputtering current of 49 mA) respectively. These powers have been selected to avoid the formation of a film on the surface of the PEEL as observed for high power conditions,^58^ while producing substantial amounts of NPs. All depositions were carried out at a pressure of 0.5 Pa without applying any intentional heating to the substrate. For all depositions, the base pressure was less than 5 × 10^−5^ Pa, and the rotation speed of the substrate was fixed to 5 rpm. The deposition time was fixed to 5 min. The deposition conditions are summarized in Table 2.

### Annealing of the NPs

A glycerol bath was heated at the desired temperature with a hot plate. A magnetic stirrer at 100 rpm was induced to homogenize the temperature of the oil bath. Then, the glass vessel containing the solution of NPs in PEEL was suspended in this bath. The growth of the NPs was achieved by annealing the PEEL solution containing the NPs for 5 hours. The size of the NPs was tuned using increasing annealing temperatures: 80, 150, and 200 °C.

### Preparation of the TEM grids

The TEM samples were prepared by dipping a copper grid with lacey carbon coating in the PEEL solution containing the NPs. The grid was heated up to 50 °C on a hot plate for 30 min over an aluminum foil. In our previous work, we demonstrated that using the same procedure, the size of the NPs obtained by TEM was similar to the one from the SAXS. So, the annealing of the grid at 50 °C here does not induce coalescence.^27^ Then, 10 μl of ethanol was dropped on the grid and dried in air at room temperature for 10 min. This step was repeated three times. Finally, the grid was dipped into ethanol for one hour, transferred into clean ethanol, and left again inside the ethanol solution for one hour. This procedure leads to the removal of excess liquid PEEL, whereas the NPs were bound to the grid. The resulting grid was then stored in a vacuum for further processing.

### Characterization

For UV-visible absorption spectroscopy (UV-vis), the PEEL solutions containing NPs were diluted in pure PEEL (1/50), poured into a 1 cm plastic cuvette, and analyzed using a Specord 250 from Analytik Jena and analysed using the software WinASPECT. The spectra were recorded between 350 and 800 nm at room temperature. The plastic cuvette filled with pure PEEL was taken as a reference. The morphology of the particles was monitored using a JEOL 2200 FS transmission electron microscope (TEM) with an acceleration voltage of 200 kV. Automated crystal orientation mapping (ACOM) was performed using a Nanomegas precession system. A precession angle of 0.63° and pixel step of 2 nm were used. The misorientation plot was obtained with ATEX software.^59^

For small-angle X-ray scattering (SAXS) analysis, the PEEL liquid containing the NPs was poured into a borosilicate capillary having an outer diameter of 1 mm and a wall thickness of 0.01 mm, as provided by WJM-Glas Müller GmbH. The SAXS measurements were performed using a Xeuss 2.0 (Xenocs) equipped with a Cu microfocus X-ray source (wavelength *λ* = 0.15418 nm). The sample to detector distance was tuned between 350 and 2500 mm to reach the q range of 0.004–149.5 Å^−1^ in a high-resolution mode (beam size 0.5 × 0.5 mm^2^). SAXS patterns measured in the transmission mode were acquired with a Pilatus 200 k (Dectris) hybrid pixel single-photon counting detector. Measured data were azimuthally integrated using Foxtrot software^60^ and corrected for background scattering from a capillary and PEEL liquid. The uncertainty of the mean radius reported in the paper is 1%.

The photoluminescence spectra were recorded on a FluoroMax-2 spectrometer from Horiba Jobin Yvon, using an excitation wavelength of 405 nm. The resulting spectra have been corrected for the background originating from PMMA semi-micro cuvettes, in which the samples have been measured.

### DFT calculation

Density Functional Theory (DFT) based total energy calculations were performed on model systems to better understand the chemical interactions between the PEEL organic oil and the different facets of the NPs. DFT calculations were carried out in a slab approach with periodic boundary conditions, as implemented in the SIESTA 4.1 code,^61^ to describe in first approximation the NP facets as flat surfaces. In the case of Au, Ag and Cu for the slab approach 5 layers were used for (111) and (110) and 6 layers for (100) in order to maintain a similar thickness at the lower layer spacing. In the case of Cu_2_O, to have an equivalent thickness, 4 layers were used for (110), 5 layers for (111) and 6 layers for (100) facets, with a Cu-termination for (110) and (100). The exchange–correlation functional is defined within the general gradient approximation using the Perdew–Burke–Ernzerhof functional.^62^ A double-ζ polarized numerical atomic basis set is adopted for the valence electrons, whereas core electrons are described with Troullier–Martin pseudopotentials.^63^ A mesh cutoff of 250 Ry and a Monkhorst–Pack grid of (2 × 2 × 1) *k*-points were used during the relaxation of the interface (PEEL molecule and the top two surface layers). These parameters were increased to 400 Ry and (4 × 4 × 1) for estimating the interaction energy. For the sake of computational facility, a simplified version of the PEEL organic oil was used by shortening the aliphatic chain to end up with the pentaerythritol molecule C(CH_2_OH)_4_. The model molecule was first fully relaxed in the isolated state using a large unit cell of (30 × 30 × 30) Å^3^ to avoid intermolecular interactions. We then studied the interactions of this model molecule with several stable and representative surfaces for a given metal or oxide, *i.e.*, the (111), (110) and (100) facets of silver, copper, copper oxide and gold NPs. Further details about the model surfaces are provided in the ESI.† The geometry relaxation was performed using the conjugated gradient formalism with a 0.04 eV Å^−1^ convergence criterion on the atomic forces. Only the molecule and the top two surface layers were allowed to relax. The interaction energy was calculated on the final relaxed geometry using the expression:*E*_int_ = *E*_surf/PEEL_ − [*E*_PEEL_ + *E*_surf_]where *E*_surf/PEEL_ is the total energy of the full interface in its optimized geometry and *E*_PEEL_ and *E*_surf_ are the energy of the free molecule and the bare surface in their interface geometry, respectively.

The charges and effective bond orders have been computed from the final DFT charge density using the DDEC/6 charge partition formalism.^64,65^ To evaluate the binding of the simplified PEEL molecule to the surface, we defined the total bond order (total BO) as the sum of the bond order of all atoms of the simplified PEEL molecule with the surface atoms. The interaction energy values, charges, and bond orders are summarized in Table 1. This approach has already been used by us to investigate the interaction of the PEEL molecule on titanium and silver oxide,^25^ and gold, gold/copper, and pure and oxidized copper (111) surfaces.^27^

## Author contributions

Adrien Chauvin: conceptualization, methodology, investigation, formal analysis, resources, visualization, writing – original draft. Anastasiya Sergievskaya: investigation, validation, methodology, formal analysis, data curation, writing – original draft. Anna Fucikova: investigation, data curation, validation. Cinthia Antunes Corrêa: investigation, data curation, validation, methodology. Jozef Vesely: investigation, data curation, validation, methodology, formal analysis, software. Jérôme Cornil: investigation, data curation, validation, methodology, formal analysis, software. David Cornil: investigation, data curation, validation, methodology, formal analysis, software. Milan Dopita: supervision, funding acquisition. Stephanos Konstantinidis: conceptualization, methodology, investigation, supervision, project administration, writing – original draft.

## Conflicts of interest

There are no conflicts to declare.

## Supplementary Material

NA-003-D1NA00222H-s001
