# Supplementary material for: Insights into the growth of nanoparticles in liquid polyol by thermal annealing
Source: Nanoscale Adv. 2021 Jun 28;3(16):4780–9. doi: 10.1039/d1na00222h (PMC9418955; doi:10.1039/d1na00222h)
Supplement: NA-003-D1NA00222H-s001 [file NA-003-D1NA00222H-s001.pdf]

Electronic Supplementary Material (ESI) for Nanoscale Advances.
This journal is © The Royal Society of Chemistry 2021

Electronic Supporting Information for:

Insights into the growth of nanoparticles in liquid polyol by thermal annealing

Adrien Chauvin,[a,b*] Anastasiya Sergievskaya,[b] Anna Fucikova,[c] Cinthia Antunes Corrêa,[d,e] Jozef Vesely,[e] Jerome Cornil,[f] David Cornil,[f] Milan Dopita,[a] and Stéphanos Konstantinidis[b]

a. Department of Condensed Matter Physics, Faculty of Mathematics and Physics, Charles University, Ke Karlovu 5, 121 16 Praha 2, Czech Republic.

b. Chimie des Interactions Plasma-Surface (ChIPS), CIRMAP, Research Institute for Materials Science and Engineering, University of Mons, 23 Place du Parc, B-7000 Mons, Belgium

c. Department of Chemical Physics and Optics, Faculty of Mathematics and Physics, Charles University, Ke Karlovu 5, 121 16 Praha 2, Czech Republic.

d. Institute of Physics of the Czech Academy of Sciences, Cukrovarnická 10/112, 162 00 Prague 6, Czech Republic.

e. Department of Physics of Materials, Faculty of Mathematics and Physics, Charles University, Ke Karlovu 5, 121 16 Praha 2, Czech Republic.

f. Laboratory for Chemistry of Novel Materials (CMN), University of Mons, Place du Parc 20, Mons 7000, Belgium.

Corresponding authors: adrien.chauvin@umons.ac.be

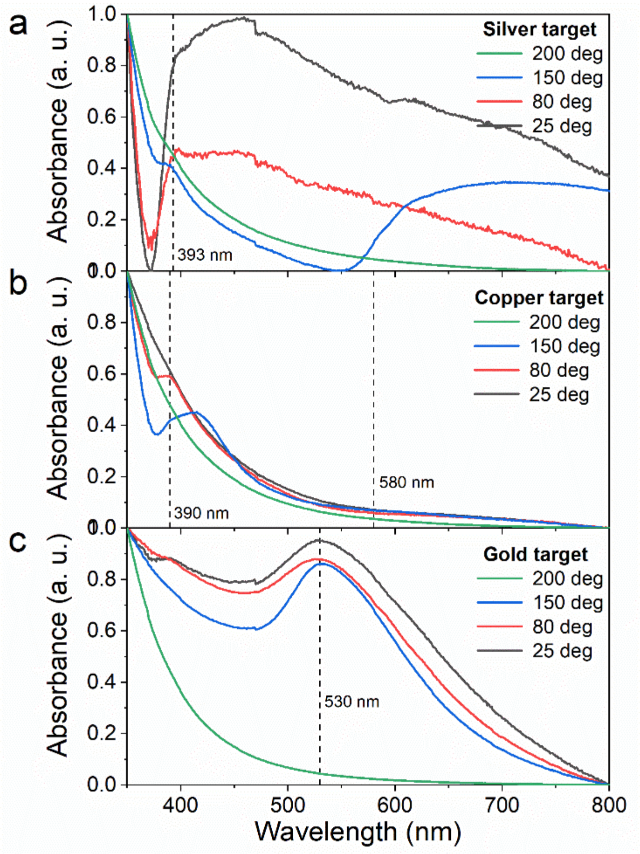

Figure S1. Absorption spectra in absolute intensity scale of diluted PEEL solution after sputtering of the (a) silver target, (b) copper target, (c) and gold target in their pristine state (black curve) and after annealing at 80 °C (red), 150 °C (blue) and 200 °C (green) during 5h.

Supplementary information 1: Details of the DFT calculation

Generation of model surfaces

The pure and oxidized metal surfaces representing different facets of nanoparticles were generated from the bulk using orthogonal unit cells, with enough surface area to prevent intermolecular interactions between PEEL molecules on the surface. The face centered cubic structure with lattice parameters used to model a N-layer slab exposing the desired facet surface and its lattice parameters a and b are summarized in Table S1.

Nature of the surface	*a* (bulk), Å	Facet	Number of layers	*a*, Å	*b*, Å
Au	4.23	111	5	11.96	10.37
		110	5	12.69	11.96
		100	6	12.69	12.69
Ag	4.09	111	5	11.55	10.00
		110	5	12.56	11.84
		100	6	12.56	12.56
Cu	3.73	111	5	10.54	13.70
		110	5	11.18	10.54
		100	6	11.18	11.18
Cu_2O	4.45	111	5	12.58	10.89
		110	4	13.34	12.58
		100	5	13.34	13.34

Table S1.

Relaxed interface geometry

Relaxed interface geometries for the pentaerythritol molecule (PE, a simplified version of PEEL) on the various surfaces considered in the manuscript can be found below.

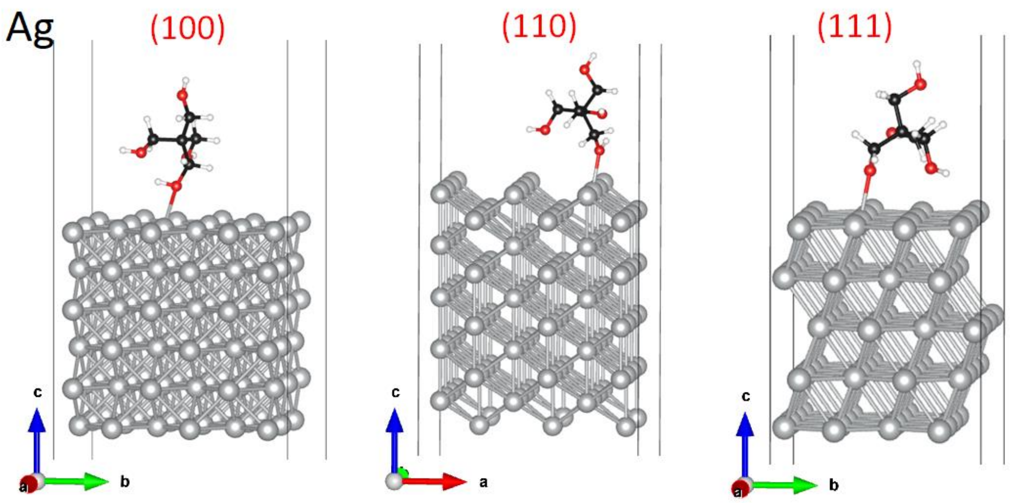

Figure S2. Side view of the final relaxed structure for PE adsorbed on Ag (100), (110) and (111) facets.

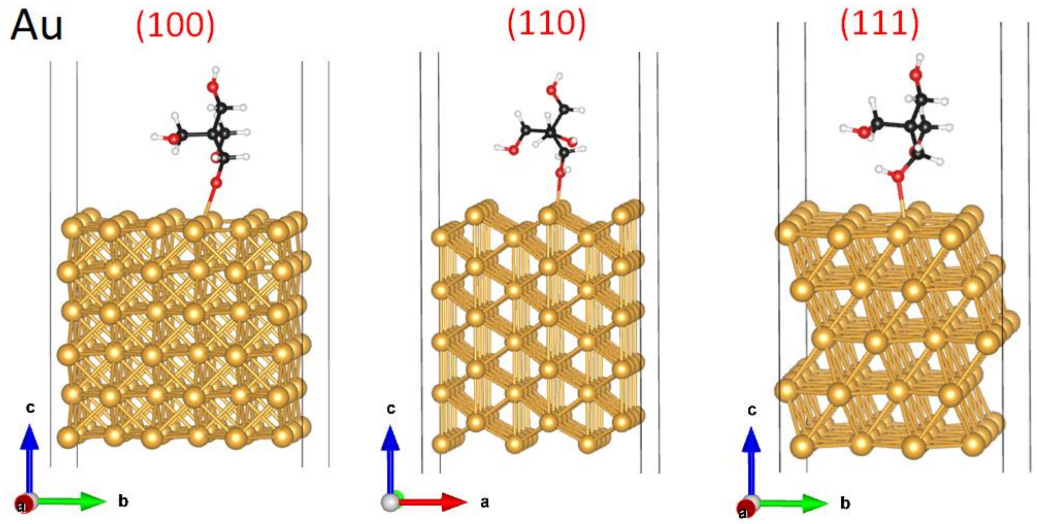

Figure S3. Side view of the final relaxed structure for PE adsorbed on Au (100), (110) and (111) facets.

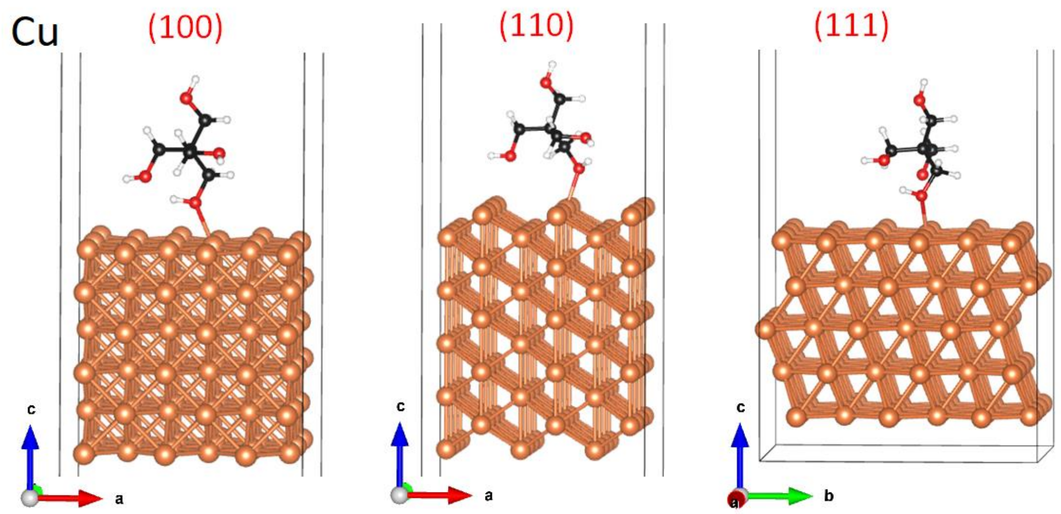

Figure S4. Side view of the final relaxed structure for PE adsorbed on Cu (100), (110) and (111) facets.

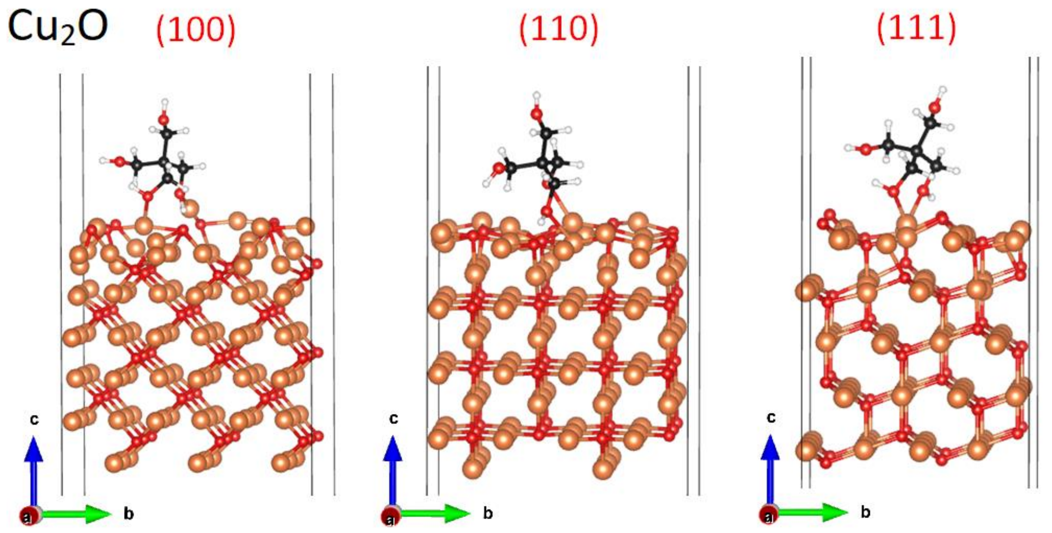

Figure S5. Side view of the final relaxed structure for PE adsorbed on Cu2O (100), (110) and (111) facets.
